# Modified Fields-Backofen and Zerilli-Armstrong constitutive models to predict the hot deformation behavior in titanium-based alloys

**DOI:** 10.1038/s41598-024-58568-9

**Published:** 2024-04-10

**Authors:** Abdallah Shokry

**Affiliations:** Department of Mechanical Engineering, Faculty of Engineering, Fayoum University, Fayoum, 63514 Egypt

**Keywords:** Hot deformation, Modified Fields-Backofen, Modified Zerilli-Armstrong, Constitutive modeling, Titanium-based alloys, Engineering, Materials science

## Abstract

This work presents modifications for two constitutive models for the prediction of the flow behavior of titanium-based alloys during hot deformation. The modified models are the phenomenological-based Fields-Backofen and the physical-based Zerilli-Armstrong. The modifications are derived and suggested by studying the hot deformation of titanium-based alloy Ti55531. The predictability of the modified models along with the original Fields-Backofen and another modified Zerilli-Armstong models is assessed and evaluated using the well-known statistical parameters correlation coefficient (R), Average Absolute Relative Error (AARE), and Root Mean Square Error (RMSE), for the Ti55531 alloy, and validated with other two different titanium-based alloys SP700 and TC4. The results show that the modified Fields-Backofen gives the best performance with R value of 0.996, AARE value of 3.34%, and RMSE value of 5.64 MPa, and the improved version of the modified Zerilli-Armstrong model comes in the second-best place with R value of 0.992, AARE value of 3.52%, and RMSE value of 9.15 MPa for the Ti55531 alloy.

## Introduction

Titanium-based alloys possess superior mechanical properties such as high strength and high corrosion resistance, especially at elevated temperatures^1–5^; hence they have been widely used in many different applications such as the aerospace industry^6,7^. Undoubtedly, dynamic recovery and dynamic recrystallization affect the microstructure as well as the mechanical properties of the titanium-based alloys during hot working such as hot rolling and hot forging^8–10^. Therefore, finding appropriate and precise models is essential for the accurate prediction of the flow behavior during hot deformation, to optimize hot working parameters such as strain, strain rate, and temperature, as well as to simulate both thermoforming processes and real applications^11–15^.

To predict the flow behavior, several constitutive models have been utilized which can be classified into three different categories namely phenomenological-based, physical-based, and artificial neural network models^16,17^. The phenomenological-based models mainly depend on empirical observations of stresses and strains such as Khan-Huang-Liang^18^, Johnson–Cook^19^ and Fields-Backofen^20^ models, while the physical-based models are mostly based on dislocation and dynamic softening phenomena during plastic deformation such as Zerilli-Armstrong^21^, Goetz-Seetharaman^22^ and Preston-Tonks-Wallace^23^ models, and the artificial neural network models uses artificial intelligence^24–27^. Titanium-based alloys have complex hot deformation behavior; therefore, constitutive models sometimes fail to accurately predict the flow behavior. Accordingly, different modifications for many constitutive models have been presented to precisely predict the flow behavior of titanium-based alloys during hot deformation^28–31^.

Fields-Backofen (FB)^20^ is one of the famous phenomenological models that is used for the prediction of the hot flow behavior. However, the model does not take thermal softening into account; therefore, it gives accurate predictions for some alloys^32–34^ and not accurate predictions for other alloys^35,36^. Therefore, some modifications for the FB model are presented that takes the thermal softening into account^31,37,38^. One of the well-known physically based models is the Zerilli-Armstrong (ZA) model^21^. In fact, the original ZA model does not take deformation parameters into account; therefore, an accurate prediction for the flow behavior is not certified, and some modifications are introduced^39–42^. Samantaray et al.^43^ introduced one of the famous modifications for the ZA model, in which the coupled effect between temperature and both strain and strain rate are considered. Very good statistical results with R = 0.995 and AARE = 5.3% are obtained when the predicted stresses are compared to experimental stresses for Ti-modified austenitic stainless steel. The modified model that was presented by Samantaray et al.^43^ was implemented to predict the hot deformation behavior for different alloys with accurate predictions^44–46^ and without accurate predictions^47–50^.

In this work, two modified constitutive models for the FB and ZA are established to predict the flow behavior of Ti53331 alloy during hot deformation. The Ti53331 is a metastable β titanium alloy, which considered one of the promising structural materials that has preference fracture toughness and outstanding fatigue properties beside the high strength^51,52^. The predictability of both modified models is compared with other modified ZA and the original FB models for the Ti55531 alloy, and evaluated using the well-known statistical parameters R, AARE, and RMSE. In addition, the modified models are validated by considering the predictability of other two different titanium-based alloys SP700 and TC4.

## Material and methods

In a recently published article, Xiang et al.^53^ studied the microstructural evolvement and dynamic softening processes of Ti55531 alloy during hot deformation in the α + β phase region. A group of experiments with different combinations of strain rates 0.001 s^−1^, 0.01 s^−1^, 0.1 s^−1^, and 1 s^−1^ and different temperatures 760 °C, 790 °C, 820 °C, and 840 °C was conducted using Gleeble-3800 test machine. For more information about the preparation of the tested samples, see reference^53^.

The hot deformation behavior of the Ti55531 alloy was found to be like the behavior of most alloys during hot deformation, in which stress increases with the increase of strain rate and the decrease of temperature. Generation and multiplication of dislocations play an important role in this increment since high stresses are needed due to the presence of dislocation interactions. Conversely, dynamic recovery and dynamic recrystallization will not find enough time to be restored^54,55^. On the other hand, stress decreases with the increase of temperature and the decrease of strain rate, which might be associated to the available enough time for dynamic recovery and dynamic recrystallization to be initiated^56,57^.

### Constitutive models

In this section, the original FB and modified ZA models as well as the presented modifications for the FB and the modified ZA models are explained.

### Fields-Backofen model (FB)

In 1957, Fields and Backofen^20^ presented their famous phenomenological model for the prediction of the hot deformation behavior. One of the drawbacks of the FB model is that it does not take the softening term into account. The FB model is given as:

1 $$\sigma = K\;\varepsilon^{n} \;\varepsilon^{ \cdot m}$$

where $\sigma$ and $\varepsilon$ represent stress and strain respectively. Constant $K$ represents strength coefficient, and constant $n$ represents the work hardening exponent. The strain rate sensitivity is presented by constant $m$. Constants $K$, $n$, and $m$ are empirically presented as next ^31,32,58^:

2 $$K = K_{0} + K_{1} \ln \varepsilon^{ \cdot } + K_{2} /T$$

3 $$n = n_{0} + n_{1} \ln \varepsilon^{ \cdot } + n_{2} /T$$

4 $$m = m_{0} + m_{1} /T$$

where $K_{0} ,\; K_{1} , \;K_{2} , \;n_{0} ,\; n_{1} , \;n_{2} , \;m_{0}$ and $m_{1}$ are material constants, which can be determined using experimental data as it will be explained later.

### Modified Zerilli-Armstrong model (MZA)

Samantaray et al.^43^ presented a familiar modification for the physical-based ZA model, to predict the hot deformation behavior, in which the coupled effect between temperature and both strain and strain rate are considered. The MZA is given as:

5 $$\sigma = \left( {C_{1} + C_{2} \varepsilon^{N} } \right)\exp \left\{ { - \left( {C_{3} + C_{4} \varepsilon } \right)T^{*} + \left( {C_{5} + C_{6} T^{*} } \right)\ln \varepsilon^{ \cdot *} } \right\}$$

The stress is represented by $\sigma$ and strain is given by $\varepsilon$, and $C_{1} , \;C_{2} , \;C_{3} , \;C_{4} ,\; C_{5} ,\; C_{6}$ and $N$ are material constants. $\varepsilon^{ \cdot *} = \varepsilon^{ \cdot } /\varepsilon_{^\circ }^{ \cdot }$ represents a value of strain rate $\varepsilon^{ \cdot }$ over a selected reference strain rate value $\varepsilon_{^\circ }^{ \cdot }$, while $T^{*} = T - T_{r}$, with $T$ represents tested temperature and $T_{r}$ introduces a selected reference temperature. In this modification, constants $C_{1} , \;C_{2}$ and $N$ represent strain hardening term, and constants $C_{3}$ and $C_{4}$ stand for softening term, while constants $C_{5}$ and $C_{6}$ constitute strain rate term.

### Modified Fields-Backofen model (MFB)

After adding softening term to the FB model and replacing strain rate with dimensionless strain rate $\varepsilon^{ \cdot } /\varepsilon_{^\circ }^{ \cdot }$ as previously defined, the proposed modification of the Fields and Backofen model can be given as next:

6 $$\sigma = K\varepsilon^{n\left( \varepsilon \right)} \left( {{\raise0.7ex\hbox{${\varepsilon^{ \cdot } }$} \!\mathord{\left/ {\vphantom {{\varepsilon^{ \cdot } } {\varepsilon_{^\circ }^{ \cdot } }}}\right.\kern-0pt} \!\lower0.7ex\hbox{${\varepsilon_{^\circ }^{ \cdot } }$}}} \right)^{{m\left( {\varepsilon ,\varepsilon^{ \cdot } } \right)}} \left( {{\raise0.7ex\hbox{${\text{T}}$} \!\mathord{\left/ {\vphantom {{\text{T}} {T_{r} }}}\right.\kern-0pt} \!\lower0.7ex\hbox{${T_{r} }$}}} \right)^{{D\left( {\varepsilon ,\varepsilon^{ \cdot } ,T} \right)}}$$

where $K$, $n$, $m$, and $D$ are material parameters, which can be determined as functions of strain, strain rate, and temperature as next:

(i) Using reference strain rate and reference temperature, Eq. (6) reduces to:

7 $$\sigma = K\varepsilon^{n\left( \varepsilon \right)}$$

Constant $K$ and parameter $n\left( \varepsilon \right)$ can be determined using Eq. (7) and experimental data at reference conditions.

(ii) At reference temperature and using $\varepsilon^{ \cdot *} = \varepsilon^{ \cdot } /\varepsilon_{^\circ }^{ \cdot }$, Eq. (6) lowers to:

8 $$\sigma = K\varepsilon^{n\left( \varepsilon \right)} \left( {\varepsilon^{ \cdot *} } \right)^{{m\left( {\varepsilon ,\varepsilon^{ \cdot } } \right)}}$$

Taking logarithm for both sides, and after performing some rearrangements, parameter $m\left( {\varepsilon ,\varepsilon^{ \cdot } } \right)$ in Eq. (8) can be expressed as:

9 $$m\left( {\varepsilon ,\varepsilon^{ \cdot } } \right) = \ln \frac{\sigma }{{K\varepsilon^{n\left( \varepsilon \right)} }}/\ln \varepsilon^{ \cdot *}$$

Parameter $m\left( {\varepsilon ,\varepsilon^{ \cdot } } \right)$ can be determined using Eq. (9) and experimental data at reference temperature.

(iii) Using $n\left( \varepsilon \right)$ that is obtained from Eq. (7) and $m\left( {\varepsilon ,\varepsilon^{ \cdot } } \right)$ that is obtained from Eq. (9), and after performing some rearrangement, parameter $D\left( {\varepsilon ,\varepsilon^{ \cdot } ,T} \right)$ in Eq. (6) can be written as:

10 $$D\left( {\varepsilon ,\varepsilon^{ \cdot } ,T} \right) = \ln \frac{\sigma }{{K\varepsilon^{n\left( \varepsilon \right)} \times \left( {\varepsilon^{ \cdot *} } \right)^{{m\left( {\varepsilon ,\varepsilon^{ \cdot } } \right)}} }}/{\text{ln }}\left( {{\raise0.7ex\hbox{$T$} \!\mathord{\left/ {\vphantom {T {T_{r} }}}\right.\kern-0pt} \!\lower0.7ex\hbox{${T_{r} }$}}} \right)$$

The parameter $D\left( {\varepsilon ,\varepsilon^{ \cdot } ,T} \right)$ can be determined using Eq. (10) and experimental data at the left values of strain rates and temperatures.

### Improved version of MZA (IMZA)

The MZA model that was presented by Samantaray et al.^43^ can be improved by studying the effect of experimental data on the parameters that constitute strain hardening, softening, and strain rate. The IMZA can be expressed as follows:

11 $$\sigma = A\left( \varepsilon \right)\exp \left\{ {B\left( {\varepsilon ,T^{*} } \right)T^{*} + C\left( {\varepsilon ,T^{*} ,\varepsilon^{ \cdot *} } \right)\ln \varepsilon^{ \cdot *} } \right\}$$

where $A$, $B$, and $C$ represent material parameters that constitute strain hardening, softening, and strain rate terms respectively, and can be determined as next:

(i) At reference strain rate and reference temperature, Eq. (11) reduces to:

12 $$\sigma = A\left( \varepsilon \right)$$

where $A$ is a material parameter that constitutes strain hardening term, which can be determined using Eq. (12) and experimental data at reference conditions.

(ii) Using strain hardening term in Eq. (12), and performing some rearrangement at reference strain rate, parameter $B\left( {\varepsilon ,T^{*} } \right)$ in Eq. (11) can be introduced as:

13 $$B\left( {\varepsilon ,T^{*} } \right) = \ln \frac{\sigma }{A\left( \varepsilon \right)}/T^{*}$$

Parameter $B\left( {\varepsilon ,T^{*} } \right)$ can be determined using Eq. (13) and experimental data at reference strain rate.

(iii) Using $A\left( \varepsilon \right)$ that is obtained from Eq. (12) and $B\left( {\varepsilon ,T^{*} } \right)$ that is obtained from Eq. (13), and after performing some rearrangement at different strain rates and different temperatures, parameter $C\left( {\varepsilon ,T^{*} ,\varepsilon^{ \cdot *} } \right)$ in Eq. (11) can be presented as:

14 $$C\left( {\varepsilon ,T^{*} ,\varepsilon^{ \cdot *} } \right) = \left[ {\ln \frac{\sigma }{A\left( \varepsilon \right)} - B\left( {\varepsilon ,T^{*} } \right)T^{*} } \right]/\ln \varepsilon^{ \cdot *}$$

Parameter $C\left( {\varepsilon ,T^{*} ,\varepsilon^{ \cdot *} } \right)$ can be determined using Eq. (14) and experimental data at the left values of strain rates and temperatures.

## Results and discussion

The results and discussion section shows how material constants of the four models are determined. Also, it displays a comparison between experimental and predicted stresses by the four models. Furthermore, it presents an evaluation and assessment for the predictability of the flow behavior by the four models using statistical parameters. This is applied to the Ti55531 alloy. Finally, the MFB and IMZA are validated by evaluating and assessing the predictability of the flow behavior for SP700 and TC4 alloys.

### Determination of models’ constants

Considering experimental data, the effect of strain, strain rate, and temperature on the flow behaviour of the Ti55531 alloy are studied. Accordingly, the parameters that constitute the modified models are suggested and determined as will be explained in this subsection.

### FB model constants

Taking logarithm of Eq. (1), it turns to:

15 $$\ln \sigma = \ln K + n\ln \varepsilon + m\ln \varepsilon^{ \cdot }$$

**Figure 1:**
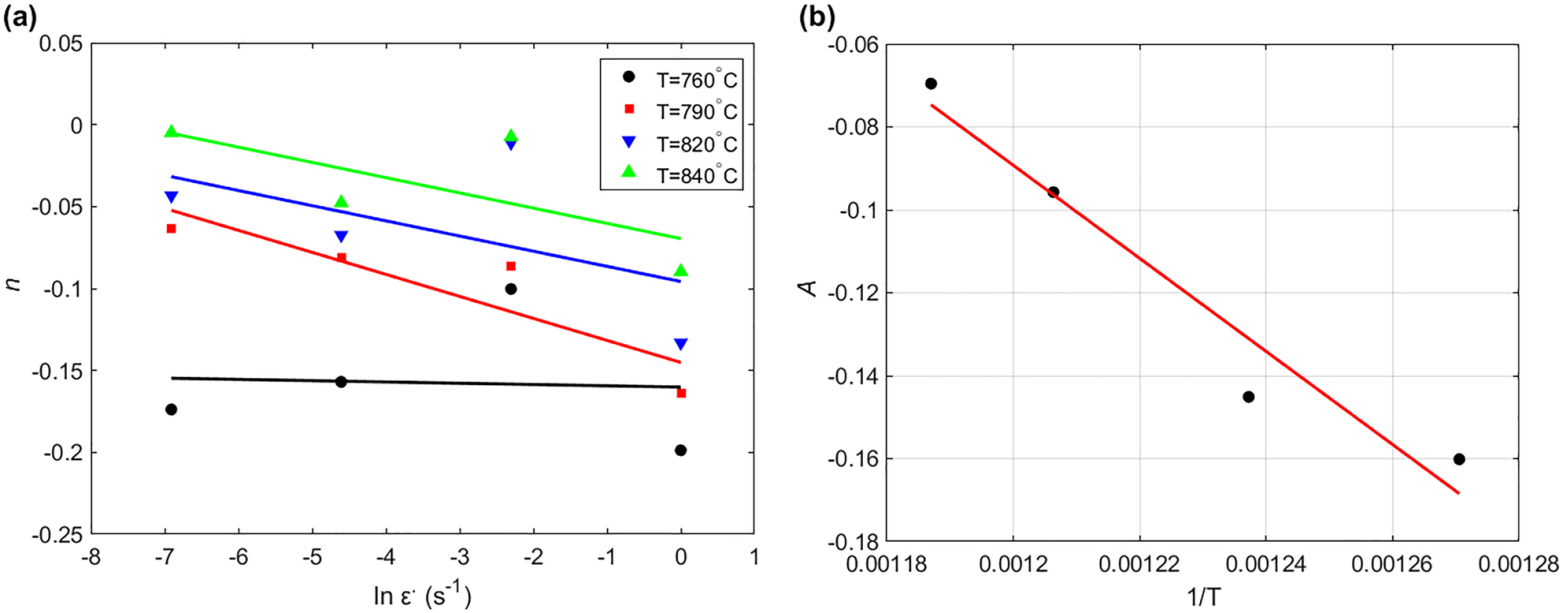
(**a**) $\ln \varepsilon^{ \cdot }$ versus $n$, and (**b**) $1/T$ versus $A$.

Considering $\ln K$ and $\ln \varepsilon^{ \cdot }$ are constants at a certain temperature. Then, $n$ is given by taking derivative of Eq. (15) as $n = {\text{d ln}}\sigma /{\text{d}} \ln \varepsilon$. By plotting ${\text{d ln}}\sigma$ versus ${\text{d}} \ln \varepsilon$, $n$ can be determined as the slope. According to the combinations of strain rate and temperature, different $n$ values are obtained. Figure 1a shows that a linear relationship between $\ln \varepsilon^{ \cdot }$ and $n$ for the four tested temperatures can be introduced as:

16 $$n = A_{1} \ln \varepsilon^{ \cdot } + A$$

The slope of the curves gives the value ${A}_{1}$, in which the average of the four values of ${A}_{1}$ can be utilized as the slope of four curves that is equal to ${n}_{1}$ in Eq. (3), which is determined as  − 0.0082. By plotting $1/T$ versus $A$ as shown in Fig. 1b, ${n}_{0}$ and $n_{2}$ in Eq. (3) are determined as 0.8162 and  − 748.32, the values of intercept and slope respectively.

**Figure 2:**
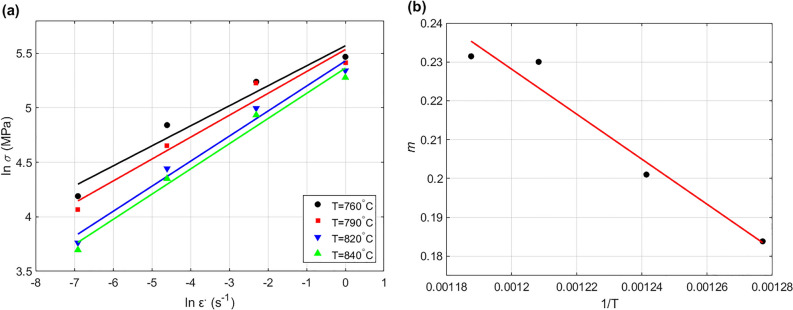
(**a**) $\ln \varepsilon^{ \cdot }$ versus $\ln \sigma$, and (**b**) $1/T$ versus $m$.

Considering $\ln K$ and $\ln \varepsilon$ are constants at a certain temperature. Then, $m$ is given by taking derivative of Eq. (15) as $m = {\text{d ln}}\sigma /{\text{d}} \ln \varepsilon^{ \cdot }$. By plotting $\ln \sigma$ versus $\ln {\upvarepsilon }^{ \cdot }$ as shown in Fig. 2a, the intercept is obtained for the four temperature values, and plotted versus $1/T$ as shown in Fig. 2b. The values of $m_{0}$ and $m_{1}$ in Eq. (4) are determined as 0.7282 and − 413.96 from the slope and intercept of the curve presented in Fig. 2b.

To obtain the constant $K$ in Eq. (2), Eq. (1) can be expressed as:

17 $$K = \frac{\sigma }{{\varepsilon^{n} \varepsilon^{ \cdot m} }}$$

**Figure 3:**
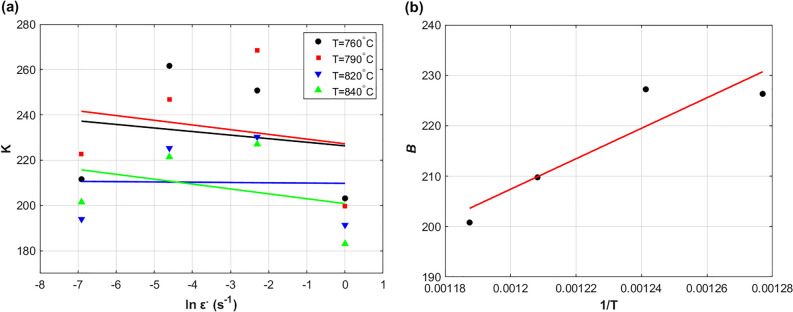
(**a**) $\ln \varepsilon^{ \cdot }$ versus $K$, and (**b**) $1/T$ versus $B$.

Substituting the obtained values of $n$ and $m$ using Eqs. (3) and (4) into Eq. (17), different $K$ values at different strain rates and different temperatures are determined. By plotting $\ln \varepsilon^{ \cdot }$ versus $K$ for the four different temperatures, the value $K_{1}$ in Eq. (2) is determined as  − 1.131 by taking the average of the slope values as shown in Fig. 3a, while both values of the parameters $K_{0}$ and $K_{2}$ are determined as  − 100.7 and 258417 respectively by plotting $1/T$ versus the intercepts ($B$) as shown in Fig. 3b.

### MZA model constants

At reference strain rate, Eq. (5) reduces to:

18 $$\sigma = \left( {C_{1} + C_{2} \varepsilon^{N} } \right)\exp \left\{ { - \left( {C_{3} + C_{4} \varepsilon } \right)T^{*} } \right\}$$

Reference strain rate is chosen as 0.001 s^−1^. Constant $C_{1}$ is determined as the yield stress with value of 90 MPa. Taking logarithm of both sides, Eq. (18) can be introduced as:

19 $$\ln \sigma = \ln \left( {C_{1} + C_{2} \varepsilon^{N} } \right) - \left( {C_{3} + C_{4} \varepsilon } \right)T^{*}$$

**Figure 4:**
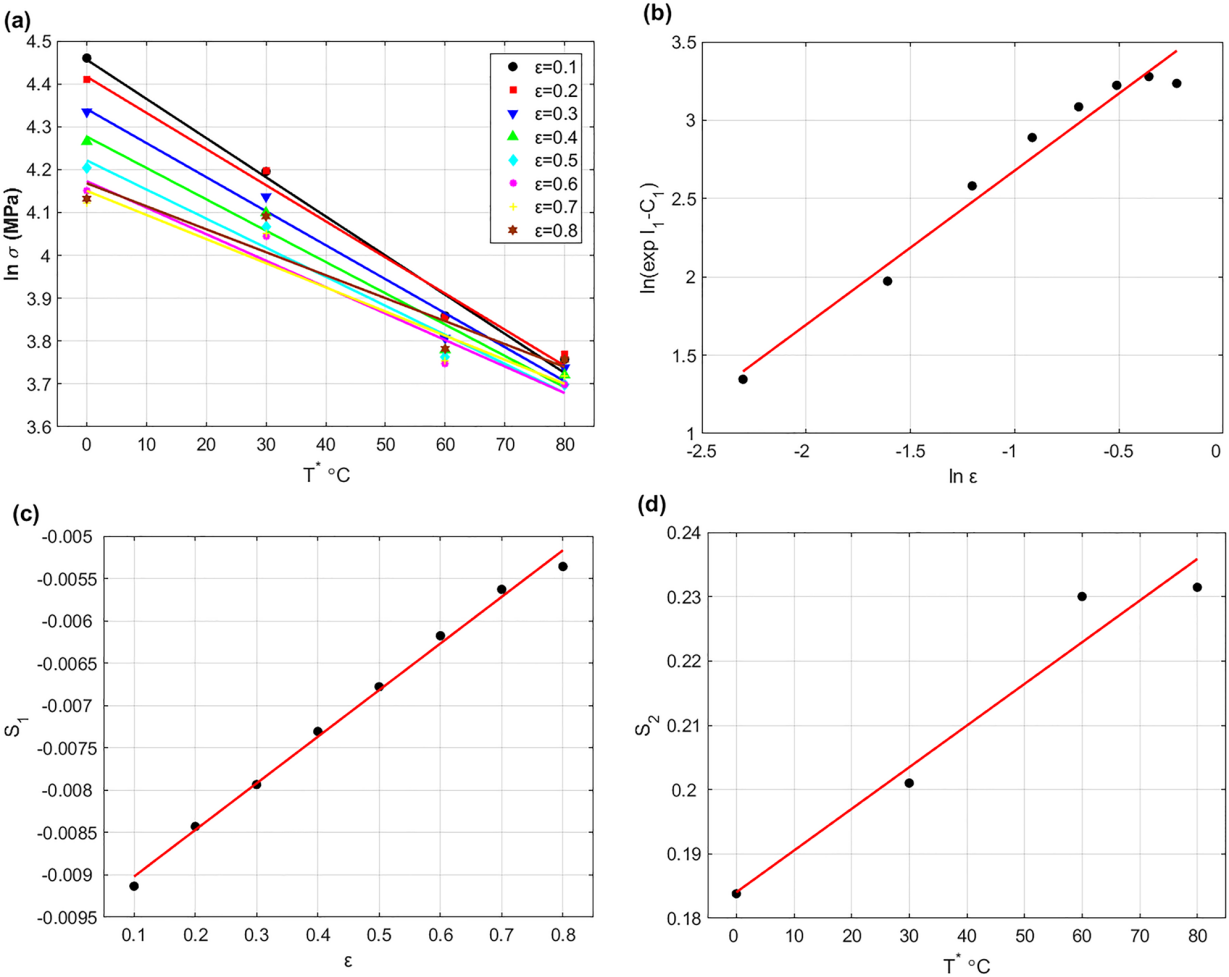
(**a**) ${T}^{*}$ versus $\ln \sigma$, (**b**) ${\text{ln}}\varepsilon$ versus $\ln \left( {\exp I_{1} - C_{1} } \right)$, (**c**) $\varepsilon$ versus ${S}_{1}$, and (**d**) ${T}^{*}$ versus ${S}_{2}$.

By plotting $T^{*}$ versus $\ln \sigma$ at reference strain rate, the slope and intercept can be determined as $- \left( {C_{3} + C_{4} \varepsilon } \right)$ and $\ln \left( {C_{1} + C_{2} \varepsilon^{N} } \right)$ respectively, for the four temperature values and with strain from 0.1 to 0.8 with an increment of 0.1 (cf. Figure 4a). To determine ${C}_{2}$, ${C}_{3}$, and ${C}_{4}$, let ${I}_{1}={\text{ln}}\left({C}_{1}+{C}_{2}{\varepsilon }^{N}\right)$ and ${S}_{1}=-\left({C}_{3}+{C}_{4}\varepsilon \right)$. The intercept ${I}_{1}$ can be rewritten in the next form after taking logarithm for both sides and performing some rearrangements:

20 $$\ln \left( {\exp I_{1} - C_{1} } \right) = \ln C_{2} + N\ln \varepsilon$$

By plotting ${\text{ln}}\varepsilon$ versus $\ln \left( {\exp I_{1} - C_{1} } \right)$ as shown in Fig. 4b, ${C}_{2}$ is determined as 38.976 from the intercept and $N$ is determined as 0.9842 from the slope. The constants ${C}_{3}$ and ${C}_{4}$ are determined as − 0.0096 and 0.0055 respectively, via plotting $\varepsilon$ versus ${S}_{1}$, in which ${C}_{3}$ represents the intercept and ${C}_{4}$ represents the slope (cf. Figure 4c). To determine ${C}_{5}$ and ${C}_{6}$, Eq. (5) can be written in the next form after taking logarithm and performing some rearrangements:

21 $$\ln \sigma = \ln \left( {C_{1} + C_{2} \varepsilon^{N} } \right) - \left( {C_{3} + C_{4} \varepsilon } \right)T^{*} + \left( {C_{5} + C_{6} T^{*} } \right)\ln \varepsilon^{ \cdot *}$$

By plotting ${\text{ln}}{\varepsilon }^{\bullet *}$ versus ${\text{ln}}\sigma$ for the left values of strain rate and temperature, ${C}_{5}+{C}_{6}{T}^{*}$ can be determined as the slope. Let ${S}_{2}={C}_{5}+{C}_{6}{T}^{*}$, and plot ${T}^{*}$ versus ${S}_{2}$, ${C}_{5}$ and ${C}_{6}$ represent the intercept and slope that are determined as 0.00065 and 0.184 respectively (cf. Figure 4d).

### MFB model constants

**Figure 5:**
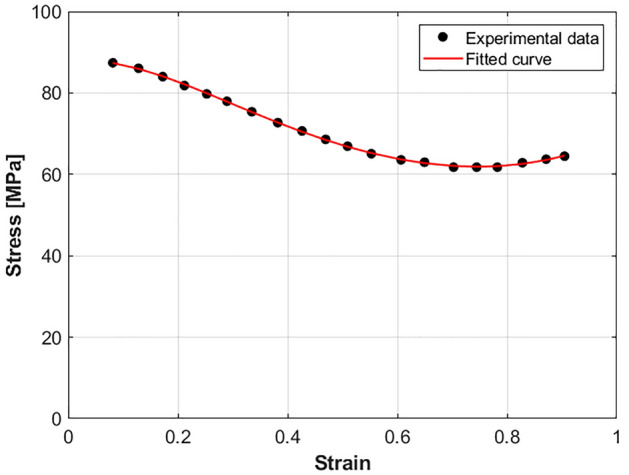
Experimental Stresses versus strains at 0.001 s^−1^ and 760 °C.

At a chosen reference strain rate of 0.001 s^−1^ and reference temperature of 760 °C, Eq. (7) can be fitted to experimental data as next (cf. Fig. 5):

22 $$\sigma = K\varepsilon^{{E_{0} + E_{1} \varepsilon + E_{2} \varepsilon^{2} }}$$

where the constants $K$, ${E}_{0}$, ${E}_{1}$,and ${E}_{2}$ are determined as 69.375 MPa,  − 0.0537,  − 0.5991, and 1.601 respectively.

**Figure 6:**
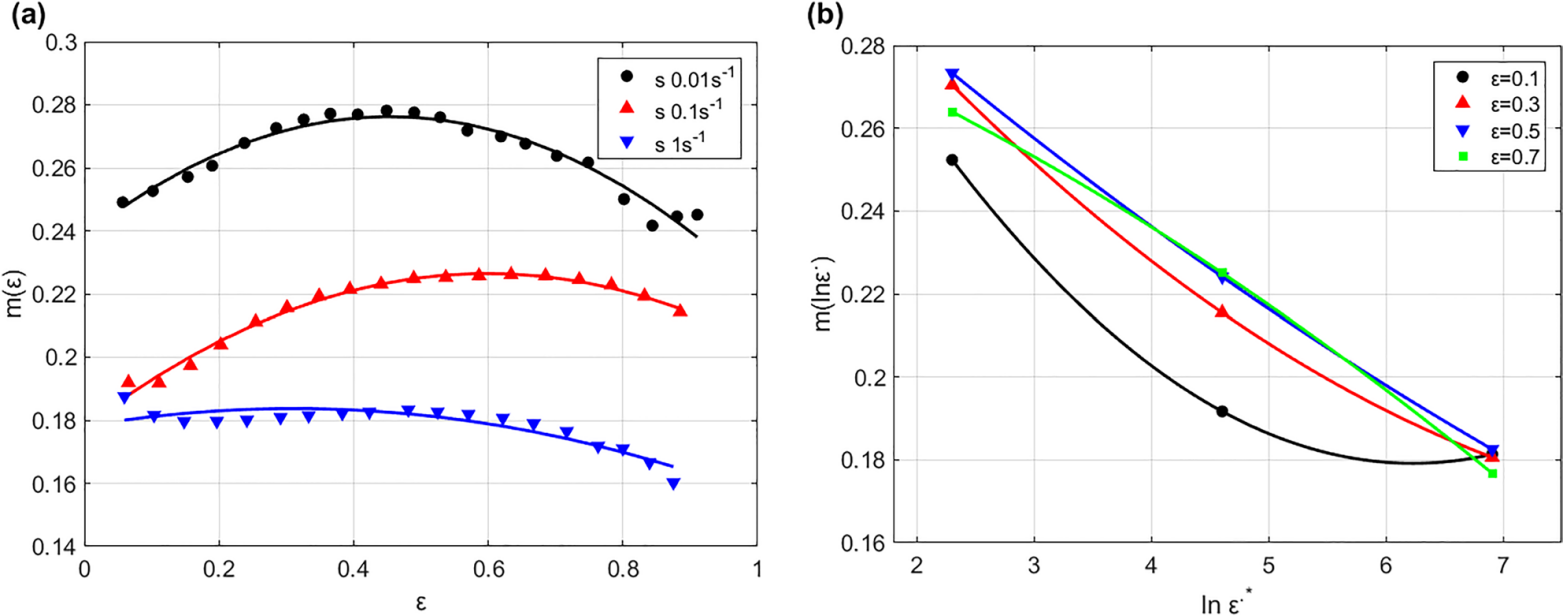
(**a**) $\varepsilon$ versus $m\left( \varepsilon \right)$ at *T* = 760 °C, and (**b**) $\ln \varepsilon^{ \cdot *}$ versus $m\left( {\ln \varepsilon^{ \cdot } } \right)$ at *T* = 760 °C.

To determine the parameter $m(\varepsilon ,{\varepsilon }^{\bullet })$ (see Eq. (9)), the effect of both strain and strain rate on $m$ at reference temperature is studied and shown in Fig. 6. As it can be seen, both strain (cf. Figure 6a) and strain rate (Fig. 6b) can be fitted to $m$ with quadratic function. Therefore, the parameter $m$ can be introduced as next:

23 $$m\left( {\varepsilon ,\varepsilon^{ \cdot } } \right) = m_{0} + m_{1} \varepsilon + m_{2} \ln \varepsilon^{ \cdot } + m_{3} \varepsilon \ln \varepsilon^{ \cdot } + m_{4} \varepsilon^{2} + m_{5} \ln \varepsilon^{ \cdot 2} + m_{6} \varepsilon^{2} \ln \varepsilon^{ \cdot 2}$$

Using Eqs. (22) and (23), Eq. (9) can be written in the next form:

24 $$\ln \frac{\sigma }{{K\varepsilon^{n\left( \varepsilon \right)} }}/\ln \varepsilon^{ \cdot *} = m_{0} + m_{1} \varepsilon + m_{2} \ln \varepsilon^{ \cdot } + m_{3} \varepsilon \ln \varepsilon^{ \cdot } + m_{4} \varepsilon^{2} + m_{5} \ln \varepsilon^{ \cdot 2} + m_{6} \varepsilon^{2} \ln \varepsilon^{ \cdot 2}$$

Although that the relationship between the output in the left side of Eq. (24) and both strain and strain rate in the right side is non-linear, the equation remains linear in the coefficients. Therefore, the constants can be determined by linear regression model that is based on least square fit using Matlab. The constants ${m}_{0}$, ${m}_{1}$, $m_{2}$, $m_{3}$, $m_{4}$, $m_{5}$, and $m_{6}$ are determined using regression analysis as 0.1769, 0.0379, 0.0029,  − 0.035,  − 0.057, 0.0032, and  − 0.007 respectively.

**Figure 7:**
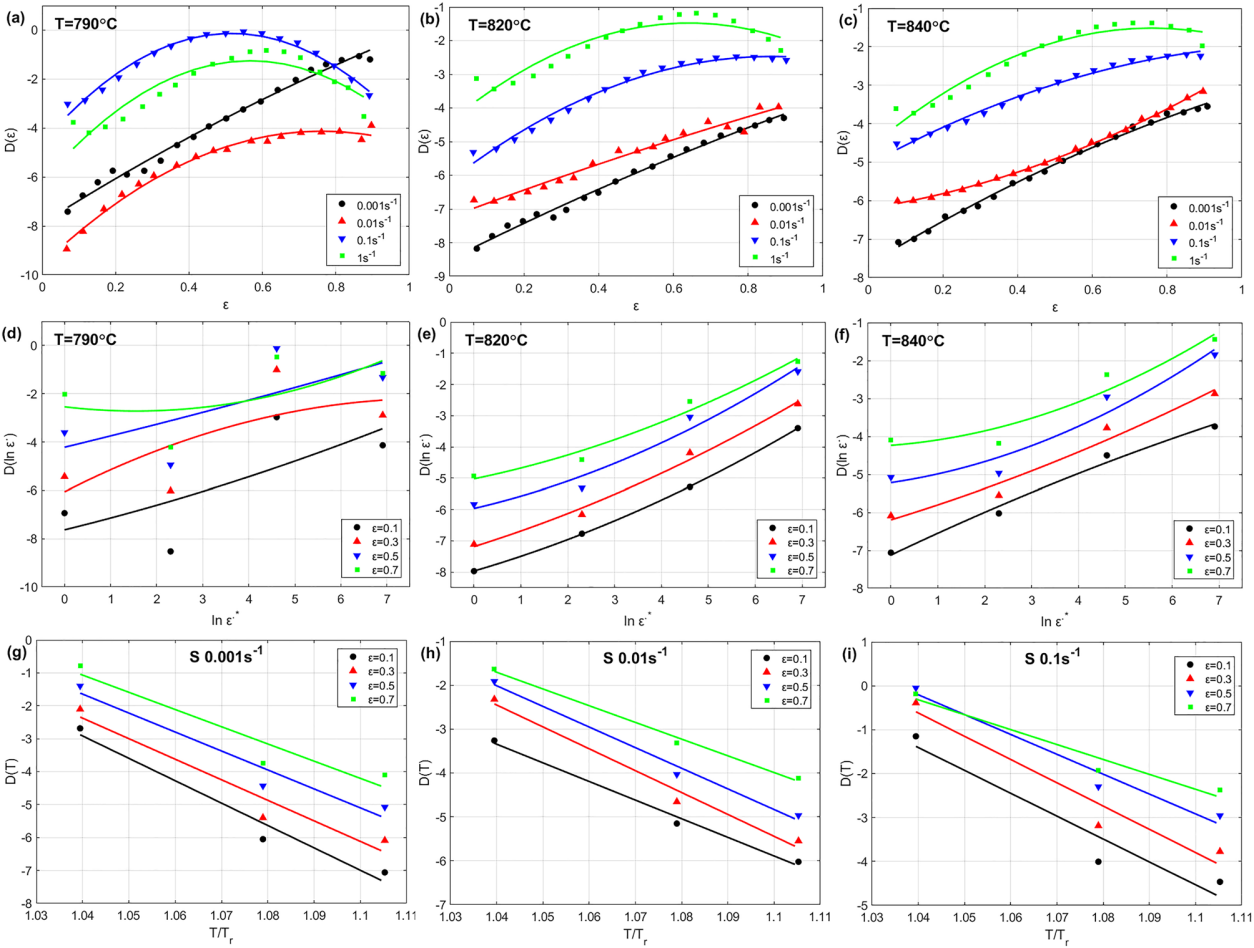
Effect of $$\varepsilon ,\varepsilon^{ \cdot }$$, and $${\raise0.7ex\hbox{$T$} \!\mathord{\left/ {\vphantom {T {T_{r} }}}\right.\kern-0pt} \!\lower0.7ex\hbox{${T_{r} }$}}$$ on $$D$$ (**a**, **b**, **c**) $$\varepsilon$$ versus of $$D\left( \varepsilon \right)$$ at 790 °C, 820 °C, and 840 °C, (**d**, **e**, **f**) $$\ln \varepsilon^{ \cdot *}$$ versus $$D\left( {\ln \varepsilon^{ \cdot } } \right)$$ at 790 °C, 820 °C, and 840 °C, and (**g**, **h**, **i**) $${\raise0.7ex\hbox{$T$} \!\mathord{\left/ {\vphantom {T {T_{r} }}}\right.\kern-0pt} \!\lower0.7ex\hbox{${T_{r} }$}}$$ versus $$D\left( T \right)$$ at 0.001 s^−1^, 0.01 s^−1^, and 0.1 s^−1^.

To determine the parameter $D\left(\varepsilon ,{\varepsilon }^{\bullet },T\right)$ (see Eq. (10)), the effect of strain, strain rate, and temperature is analyzed at the left combinations of strain rate and temperature on parameter $D$, and shown in Fig. 7. The first raw in Fig. 7 shows the effect of strain versus ${\text{D}}$ at 790 °C, 820 °C, and 840 °C, while the second raw represents the effect of strain rate on ${\text{D}}$ at 790 °C, 820 °C, and 840 °C, and finally the third raw displays the effect of temperature on ${\text{D}}$ at 0.001 s^−1^, 0.01 s^−1^, and 0.1 s^−1^. As it can be seen, quadratic fitting can be implemented with both strain and strain rate, while a linear fitting might be enough for temperature; therefore, the parameter $D\left(\varepsilon ,{\varepsilon }^{\bullet },T\right)$ can be introduced as:

25 $$D\left( {\varepsilon ,\varepsilon^{ \cdot } ,T} \right){ } = D_{0} + D_{1} \varepsilon + D_{2} \ln \varepsilon^{ \cdot } + D_{3} T + D_{4} \varepsilon \ln \varepsilon^{ \cdot } + D_{5} \varepsilon^{2} + D_{6} \ln \varepsilon^{ \cdot 2} + D_{7} \varepsilon^{2} \ln \varepsilon^{ \cdot 2}$$

Using Eqs. (22), (23) and (25), Eq. (10) can be written in the next form:

26 $$\ln \frac{\sigma }{{K\varepsilon^{n\left( \varepsilon \right)} \times \left( {\varepsilon^{ \cdot *} } \right)^{{m\left( {\varepsilon ,\varepsilon^{ \cdot } } \right)}} }}/{\text{ln }}\left( {{\raise0.7ex\hbox{$T$} \!\mathord{\left/ {\vphantom {T {T_{r} }}}\right.\kern-0pt} \!\lower0.7ex\hbox{${T_{r} }$}}} \right) = D_{0} + D_{1} \varepsilon + D_{2} \ln \varepsilon^{ \cdot } + D_{3} T + D_{4} \varepsilon \ln \varepsilon^{ \cdot } + D_{5} \varepsilon^{2} + D_{6} \ln \varepsilon^{ \cdot 2} + D_{7} \varepsilon^{2} \ln \varepsilon^{ \cdot 2}$$

Considering the same procedure as done with the determination of constants in Eq. (24), the constants ${D}_{0}$, ${D}_{1}$, ${D}_{2}$, ${D}_{3}$, ${D}_{4}$, ${D}_{5}$, ${D}_{6}$ and ${D}_{7}$ are determined using regression analysis as 8.359, 9.649, 0.557,  − 0.016, 0.239,  − 7.172,  − 0.003 and 0.106 respectively.

Finally, the MFB model can be expressed as:

27 $$\begin{array}{*{20}l} {\sigma = K\varepsilon^{n\left( \varepsilon \right)} \left( {{\raise0.7ex\hbox{${\varepsilon^{ \cdot } }$} \!\mathord{\left/ {\vphantom {{\varepsilon^{ \cdot } } {\varepsilon_{^\circ }^{ \cdot } }}}\right.\kern-0pt} \!\lower0.7ex\hbox{${\varepsilon_{^\circ }^{ \cdot } }$}}} \right)^{{m\left( {\varepsilon ,\varepsilon^{ \cdot } } \right)}} \left( {{\raise0.7ex\hbox{$T$} \!\mathord{\left/ {\vphantom {T {T_{r} }}}\right.\kern-0pt} \!\lower0.7ex\hbox{${T_{r} }$}}} \right)^{{D\left( {\varepsilon ,\varepsilon^{ \cdot } ,T} \right)}} } \hfill \\ {n\left( \varepsilon \right) = E_{0} + E_{1} \varepsilon + E_{2} \varepsilon^{2} } \hfill \\ {m\left( {\varepsilon ,\varepsilon^{ \cdot } } \right) = m_{0} + m_{1} \varepsilon + m_{2} \ln \varepsilon^{ \cdot } + m_{3} \varepsilon \ln \varepsilon^{ \cdot } + m_{4} \varepsilon^{2} + m_{5} \ln \varepsilon^{ \cdot 2} + m_{6} \varepsilon^{2} \ln \varepsilon^{ \cdot 2} } \hfill \\ {D\left( {\varepsilon ,\varepsilon^{ \cdot *} ,T} \right){ } = D_{0} + D_{1} \varepsilon + D_{2} \ln \varepsilon^{ \cdot } + D_{3} T + D_{4} \varepsilon \ln \varepsilon^{ \cdot } + D_{5} \varepsilon^{2} + D_{6} \ln \varepsilon^{ \cdot 2} + D_{7} \varepsilon^{2} \ln \varepsilon^{ \cdot 2} } \hfill \\ \end{array}$$

### IMZA model constants

**Figure 8:**
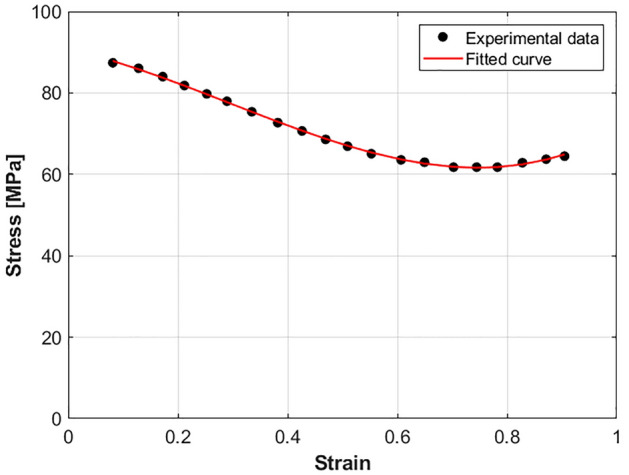
Experimental Stresses versus strains at 0.001 s^−1^ and 760 °C.

At a chosen reference strain rate of 0.001 s^−1^ and reference temperature of 760 °C, the strain hardening term in Eq. (12) can be fitted to experimental data with cubic function as shown in Fig. 8, and expressed as:

28 $$\sigma = A_{0} + A_{1} \varepsilon + A_{2} \varepsilon^{2} + A_{3} \varepsilon^{3}$$

where constants $A_{0}$, $A_{1}$, $A_{2}$, and $A_{3}$ are determined as 90.757 MPa, − 30.177, − 76.062, and 86.014 respectively.

**Figure 9:**
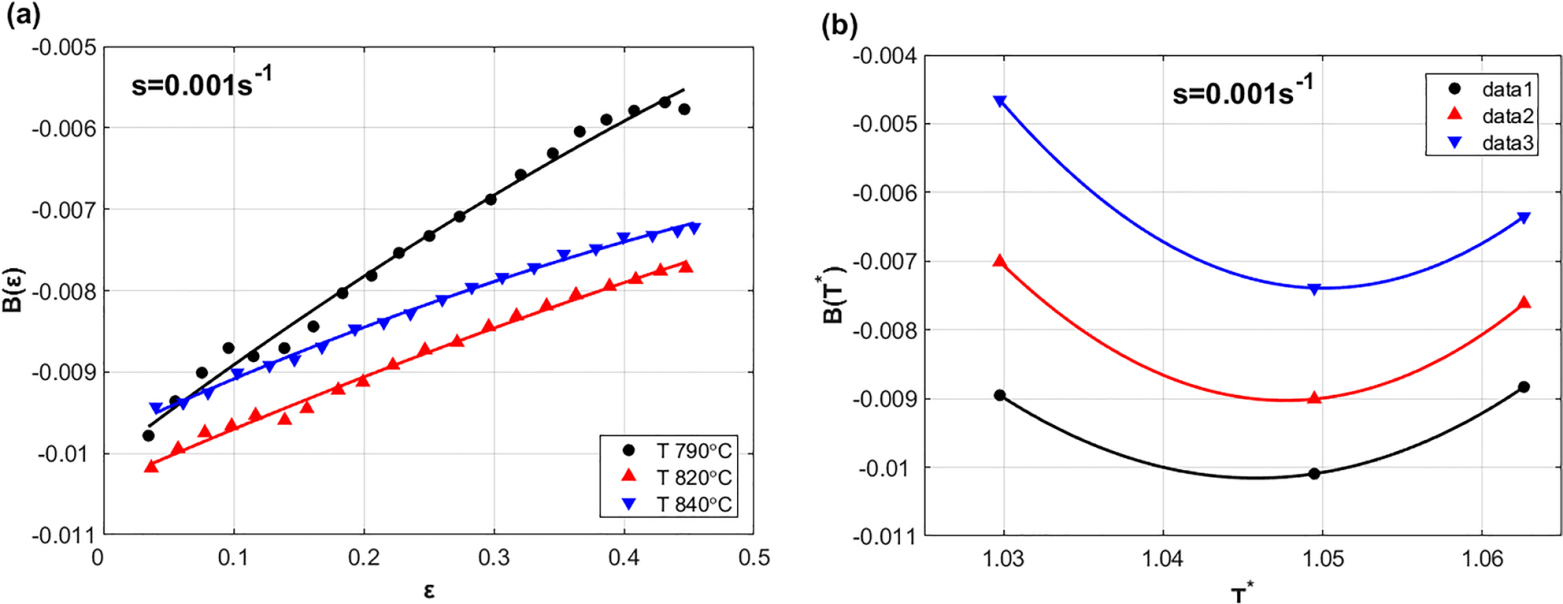
(**a**) $\varepsilon$ versus $B(\varepsilon )$ at s = 0.001 s^−1^, and (**b**) ${T}^{*}$ versus $B({T}^{*})$ at s = 0.001 s^−1^.

To determine the parameter $B\left(\varepsilon ,{T}^{*}\right)$ (see Eq. (13)), the effect of both strain and temperature on $B$ at reference strain rate is studied and shown in Fig. 9. As it can be seen, both strain (cf. Figure 9a) and temperature (cf. Figure 9b) can be fitted to $D$ with quadratic function. Therefore, the parameter $B$ can be introduced as next:

29 $$B\left( {\varepsilon ,T^{*} } \right) = B_{0} + B_{1} \varepsilon + B_{2} T^{*} + B_{3} \varepsilon T^{*} + B_{4} \varepsilon^{2} + B_{5} T^{*2} + B_{6} \varepsilon^{2} T^{*2}$$

Using Eqs. (28) and (29), Eq. (13) can be written in the next form:

30 $$\ln \frac{\sigma }{A\left( \varepsilon \right)}/T^{*} = B_{0} + B_{1} \varepsilon + B_{2} T^{*} + B_{3} \varepsilon T^{*} + B_{4} \varepsilon^{2} + B_{5} T^{*2} + B_{6} \varepsilon^{2} T^{*2}$$

Considering the same procedure of determining the constants in the previous subsection, the constants $B_{0}$, $B_{1}$, $B_{2}$, $B_{3}$, $B_{4}$, $B_{5}$ and $B_{6}$ are determined using regression analysis as − 0.0043, 0.0179, − 0.0039, − 0.0003, 2.8 × 10^−6^, − 0.0002, and 0.62 × 10^−6^ respectively.

**Figure 10:**
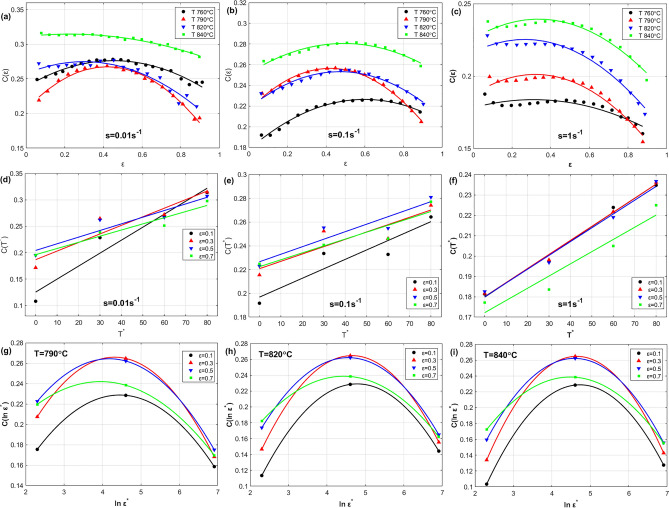
Effect of $\varepsilon ,T^{*}$, and $\varepsilon^{ \cdot *}$ on $C$ (**a**, **b**, **c**) $\varepsilon$ versus of $C\left( \varepsilon \right)$ at 0.01 s^−1^, 0.1 s^−1^, and 1 s^−1^, (**d**, **e**, **f**) $T^{*}$ versus $C\left( {T^{*} } \right)$ at 0.01 s^−1^, 0.1 s^−1^, and 1 s^−1^, and (**g**, **h**, **i**) $\normalsize ln \varepsilon^{ \cdot *}$ versus $\normalsize C\left( {ln \varepsilon^{ \cdot *} } \right)$ at 790 °C, 820 °C, and 840 °C.

To determine the parameter $C\left( {\varepsilon ,T^{*} ,\varepsilon^{ \cdot *} } \right)$ (see Eq. (14)), the effect of strain, temperature, and strain rate on parameter $C$ is analyzed at the left combinations of strain rate and temperature, and shown in Fig. 10. The first raw in Fig. 10 shows the effect of strain versus $C$ at 0.01 s^−1^, 0.1 s^−1^, and 1 s^−1^, while the second raw displays the effect of temperature on $C$ at 0.01 s^−1^, 0.1 s^−1^, and 1 s^−1^, and finally the third raw represents the effect of strain rate on $C$ at 790 °C, 820 °C, and 840 °C. As it can be seen, quadratic fitting can be implemented with both strain and strain rate, while a linear fitting might be enough for temperature; therefore, the parameter $C\left( {\varepsilon ,T^{*} ,\varepsilon^{ \cdot *} } \right)$ can be introduced as:

31 $$C\left( {\varepsilon ,T^{*} ,\varepsilon^{ \cdot *} } \right) = C_{0} + C_{1} \varepsilon + C_{2} T^{*} + C_{3} \ln \varepsilon^{ \cdot *} + C_{4} \varepsilon \ln \varepsilon^{ \cdot *} + C_{5} \varepsilon^{2} + C_{6} \ln \varepsilon^{ \cdot *2} + C_{7} \varepsilon^{2} \ln \varepsilon^{ \cdot *2}$$

Using Eqs. (28), (29) and (31), Eq. (14) can be written in the next form:

32 $$\left[ {\ln \frac{\sigma }{A\left( \varepsilon \right)} - B\left( {\varepsilon ,T^{*} } \right)T^{*} } \right]/\ln \varepsilon^{ \cdot *} = C_{0} + C_{1} \varepsilon + C_{2} T^{*} + C_{3} \ln \varepsilon^{ \cdot *} + C_{4} \varepsilon \ln \varepsilon^{ \cdot *} + C_{5} \varepsilon^{2} + C_{6} \ln \varepsilon^{ \cdot *2} + C_{7} \varepsilon^{2} \ln \varepsilon^{ \cdot *2}$$

Following the same procedure of determining constants in the previous subsection, the constants $C_{0}$, $C_{1}$, $C_{2}$, $C_{3}$, $C_{4}$, $C_{5}$, $C_{6}$ and $C_{7}$ are determined using regression analysis as 0.2415, 0.108,  − 0.0005, 0.00044, 0.005,  − 0.1478,  − 0.0015 and  − 0.0005 respectively.

Finally, the IMZA model can be expressed as next:

33 $$\begin{gathered} \sigma = A\left( \varepsilon \right)\exp \left\{ {B\left( {\varepsilon ,T^{*} } \right)T^{*} + C\left( {\varepsilon ,T^{*} ,\varepsilon^{ \cdot *} } \right)\ln \varepsilon^{ \cdot *} } \right\} \hfill \\ A\left( \varepsilon \right) = A_{0} + A_{1} \varepsilon + A_{2} \varepsilon^{2} + A_{3} \varepsilon^{3} \hfill \\ B\left( {\varepsilon ,T^{*} } \right) = B_{0} + B_{1} \varepsilon + B_{2} T^{*} + B_{3} \varepsilon T^{*} + B_{4} \varepsilon^{2} + B_{5} T^{*2} + B_{6} \varepsilon^{2} T^{*2} \hfill \\ C\left( {\varepsilon ,T^{*} ,\varepsilon^{ \cdot *} } \right) = C_{0} + C_{1} \varepsilon + C_{2} T^{*} + C_{3} \ln \varepsilon^{ \cdot *} + C_{4} \varepsilon \ln \varepsilon^{ \cdot *} + C_{5} \varepsilon^{2} + C_{6} \ln \varepsilon^{ \cdot *2} + C_{7} \varepsilon^{2} \ln \varepsilon^{ \cdot *2} \hfill \\ \end{gathered}$$

### Predicted stresses compared to experimental stresses

In this subsection, a comparison between predicted stresses by the FB, MZA, MFB, and IMZA models and experimental stresses is presented and addressed. Then, the predictability of the four models is assessed and evaluated using the well-known statistical parameters R, AARE, and RMSE.

**Figure 11:**
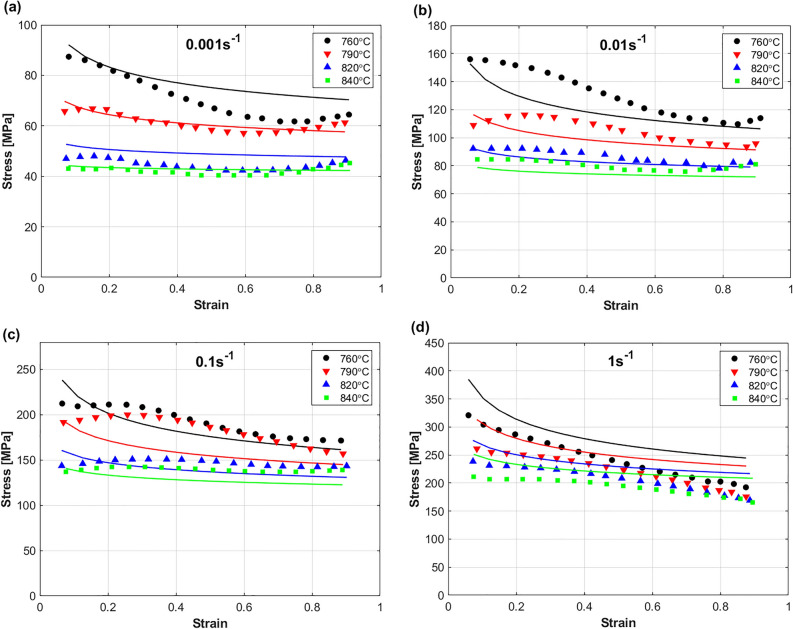
Experimental stresses (markers) compared to predicted stresses (solid lines) by FB model for TI55531 alloy at (**a**) 0.001 s^−1^, (**b**) 0.01 s^−1^, (**c**) 0.1 s^−1^, and (**d**) 1 s^−1^. Experimental data are gotten from^53^.

A comparison between predicted stresses by FB model and experimental stresses for the Ti55531 alloy during hot deformation is shown in Fig. 11. Clearly, the figure shows that the FB model failed to accurately predict the hot flow behavior of the Ti55531 alloy. A possible reason is that the FB model does not take the softening effect into account. Another possible reason is the complex behavior of the Ti55531 alloy at hot working conditions, in which the parameters $K$, n, and $m$ are simplified as linear functions in strain, strain rate, and temperature in the FB model.

**Figure 12:**
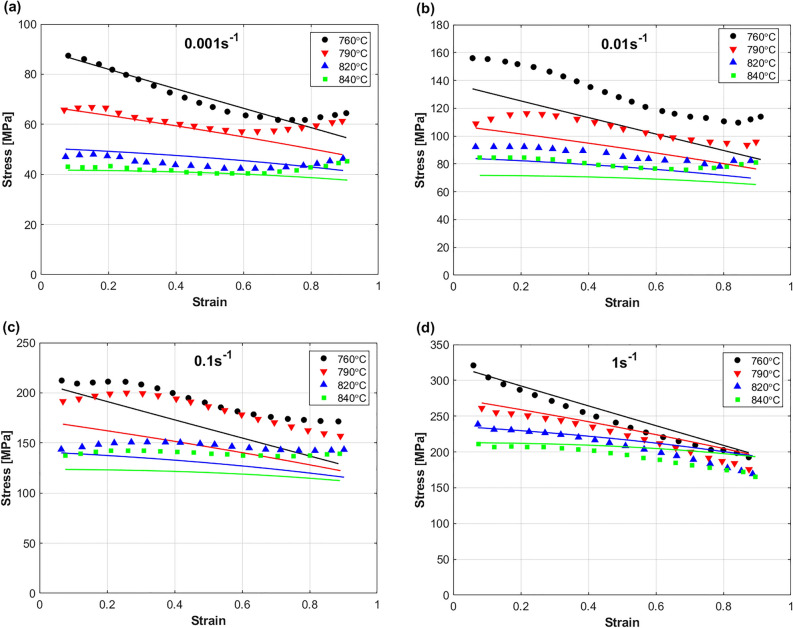
Experimental stresses (markers) compared to predicted stresses (solid lines) by MZA model for TI55531 alloy at (**a**) 0.001 s^−1^, (**b**) 0.01 s^−1^, (**c**) 0.1 s^−1^, and (**d**) 1 s^−1^. Experimental data are gotten from^53^.

Figure 12 Shows a comparison between predicted stresses by MZA model and experimental stresses of Ti55531 alloy during hot deformation. Obviously, the figure shows that the MZA model failed to accurately predict the hot flow behavior of the Ti55531 alloy. Despite that the MZA model considers the coupling effect of strain and temperature, and temperature and strain rate, this effect is simplified with linear relationship; however, the behavior of the Ti55531 alloy during hot working conditions is complex, which might be a possible reason for this lack of accuracy. Another possible reason is the coupling effect of strain, strain rate, and temperature, which had to be considered in the MZA.

**Figure 13:**
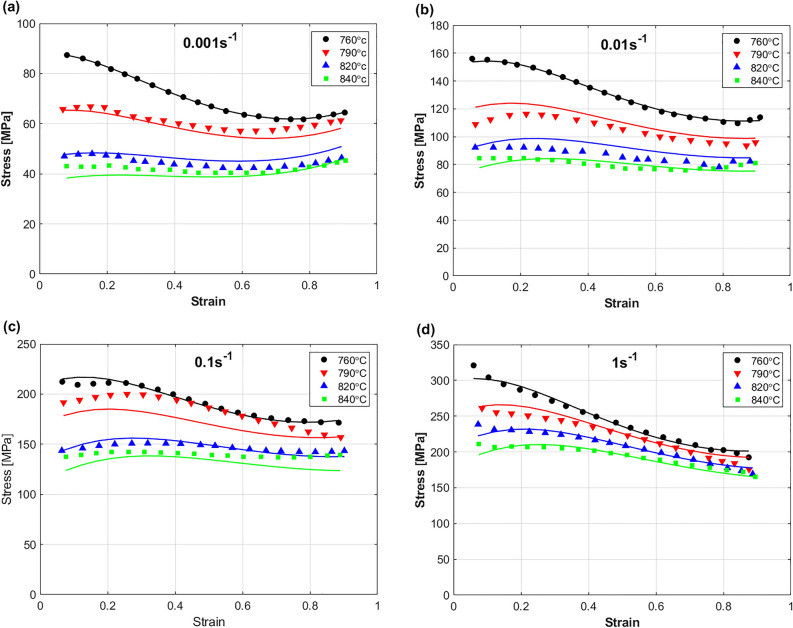
Experimental stresses (markers) compared to predicted stresses (solid lines) by MFB model for TI55531 alloy at (**a**) 0.001 s^−1^, (**b**) 0.01 s^−1^, (**c**) 0.1 s^−1^, and (**d**) 1 s^−1^. Experimental data are gotten from^53^.

A comparison between predicted stresses by MFB model and experimental stresses of the Ti55531 alloy during hot deformation is shown in Fig. 13. As it can be seen, the figure shows that the MFB model can predict the flow behavior of the Ti55531 alloy during hot deformation with a very good accuracy. Certainly, adding the softening effect to the MFB model can be one of the possible reasons for the obtained accuracy. Another reason is that the coupling effect of strain, strain rate, and temperature is considered in the MFB model. Only at strain rate of 0.1 s^−1^ and temperature of 760 °C, the MFB model could not succeed in accurately predicting the flow behavior, which might be affected by the complex non-linear behavior of the Ti55531 alloy at hot working conditions.

**Figure 14:**
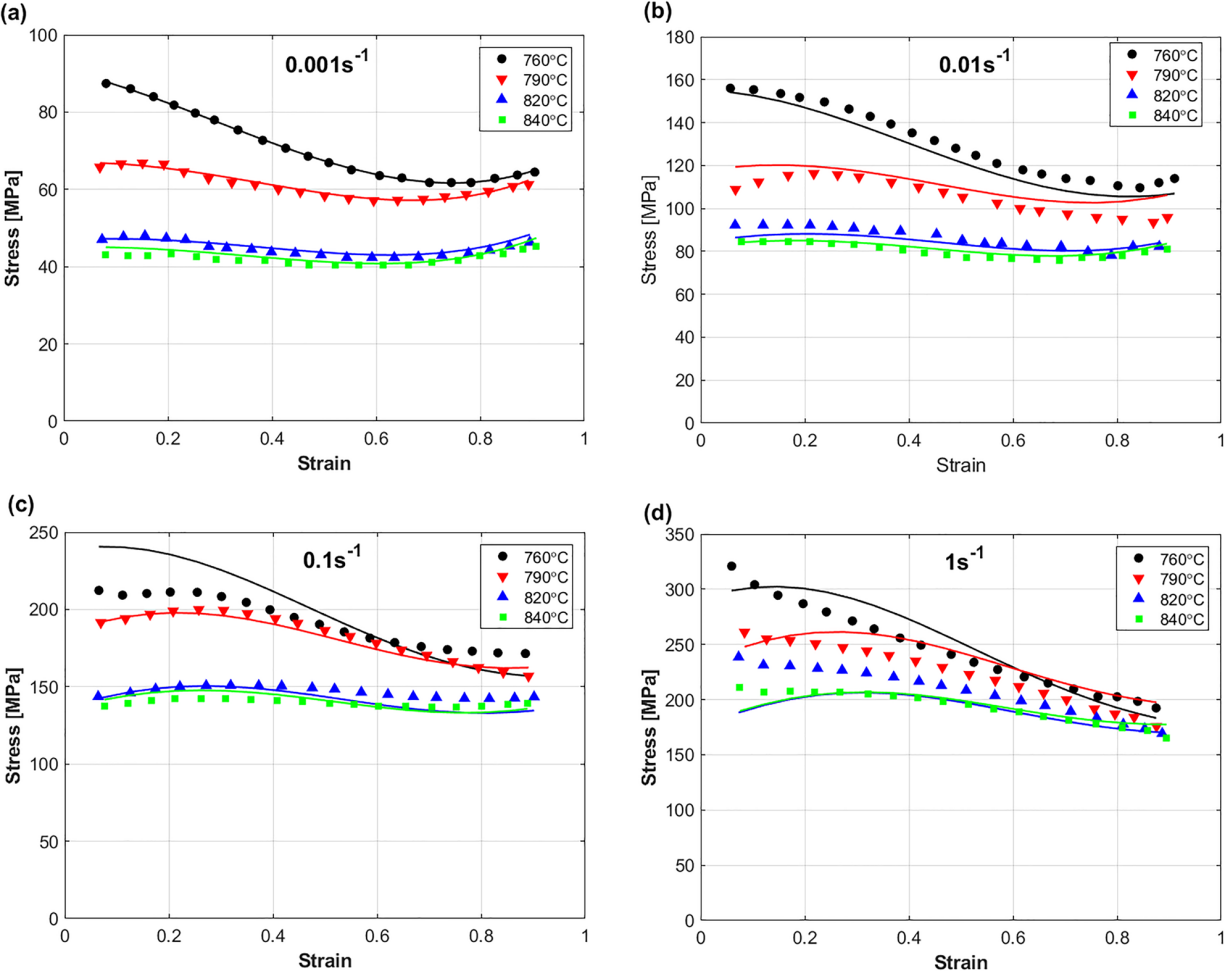
Experimental stresses (markers) compared to predicted stresses (solid lines) by IMZA model for TI55531 alloy at (**a**) 0.001 s^−1^, (**b**) 0.01 s^−1^, (**c**) 0.1 s^−1^, and (**d**) 1 s^−1^. Experimental data are gotten from^53^.

The predicted stresses obtained by IMZA model are compared to experimental stresses of the Ti55531 alloy during hot deformation as shown in Fig. 14. As it can be seen, the figure shows that the IMZA model can predict the flow behavior of the Ti55531 alloy during hot deformation with a very good accuracy at strain rates of 0.001 and 0.01 s^−1^, and with good accuracy at strain rates of 0.1 and 1 s^−1^. Taking the coupling effect between strain, strain rate, and temperature into account might be considered as one possible reason for the obtained accuracy. The complex non-linear behavior of the Ti55531 during hot working conditions might be a reason for the not very accurate predictions at strain rates of 0.1 and 1 s^−1^.

### Assessment and evaluation of the models

The predictability of the FB, MZA, MFB, and IMZA models are evaluated and assessed using the statistical parameters R, AARE, and RMSE that are computed as ^12^:

34 $$R = \frac{{\mathop \sum \nolimits_{i}^{N} \left( {\sigma_{e} - \overline{{\sigma_{e} }} } \right)\left( {\sigma_{P} - \overline{{\sigma_{P} }} } \right)}}{{\sqrt {\mathop \sum \nolimits_{i}^{N} \left( {\sigma_{e} - \overline{{\sigma_{e} }} } \right)^{2} \mathop \sum \nolimits_{i}^{N} \left( {\sigma_{P} - \overline{{\sigma_{P} }} } \right)^{2} } }}$$

35 $${\text{AARE}} = \frac{1}{N}\mathop \sum \limits_{i}^{N} \left| {\frac{{\sigma_{e} - \sigma_{P} }}{{\sigma_{e} }}} \right| \times 100$$

36 $${\text{RMSE}} = \frac{1}{N}\sqrt {\mathop \sum \limits_{i}^{N} \left( {\sigma_{e} - \sigma_{P} } \right)^{2} }$$

where $\sigma_{e}$ and $\overline{{\sigma_{e} }}$ introduces experimental stresses and its mean value, while $\sigma_{P}$ and $\overline{{\sigma_{P} }}$ represent predicted stresses and its mean value, and N introduces the total number of observations.

**Figure 15:**
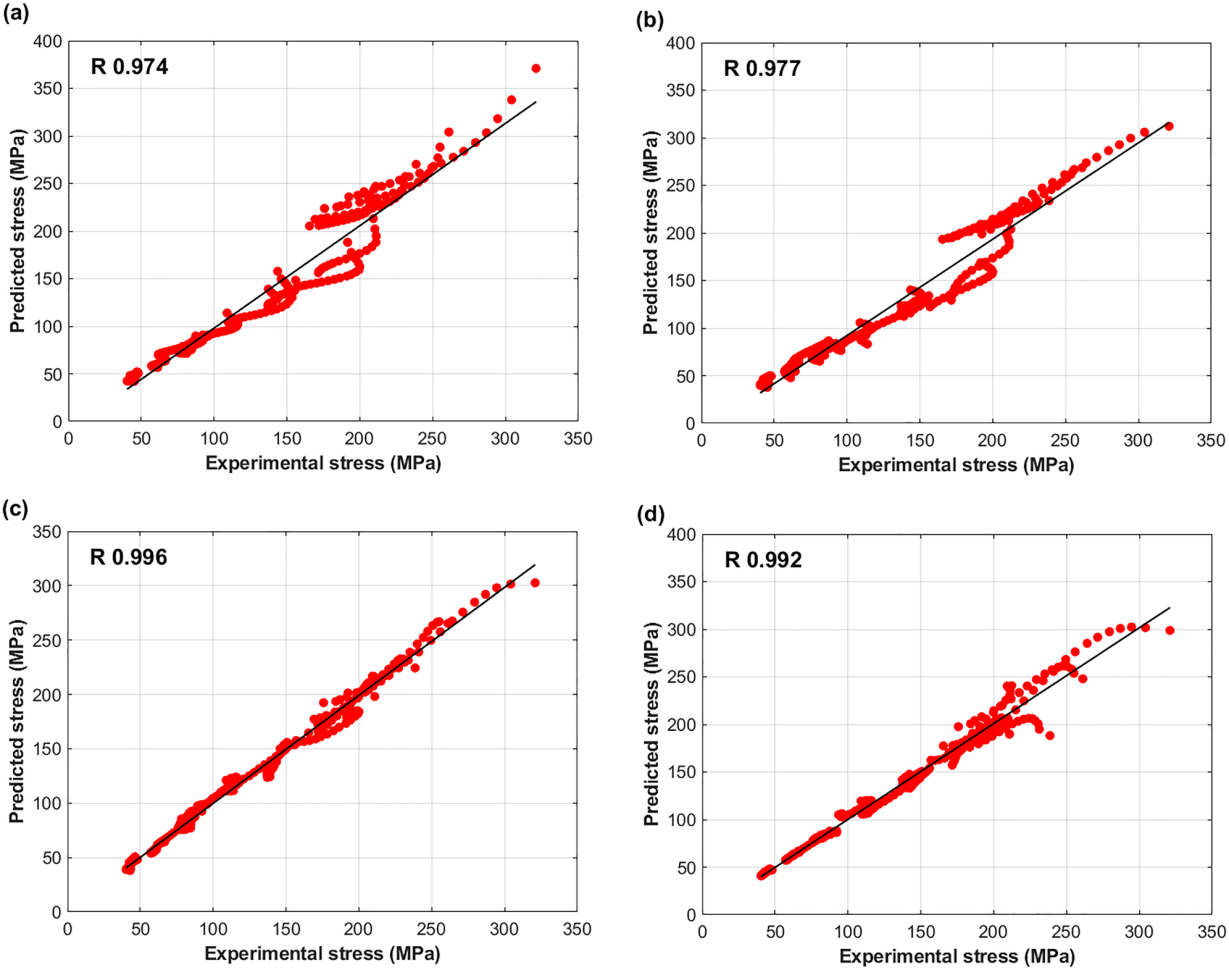
Correlation of experimental stresses and predicted stresses by (**a**) FB model, (**b**) MZA model, (**c**) MFB model, and (**d**) IMZA model.

A correlation between experimental stresses and predicted stresses by FB, MZA, MFB, and IMZA models using Eq. (34) is shown in Fig. 15. The figure shows that the MFB model gives the best $R$ value with 0.996, and the second best $R$ value is given by the IMZA model with 0.992, while both FM and MZA models give the lowest $R$ values with 0.974 and 0.977 respectively.

**Figure 16:**
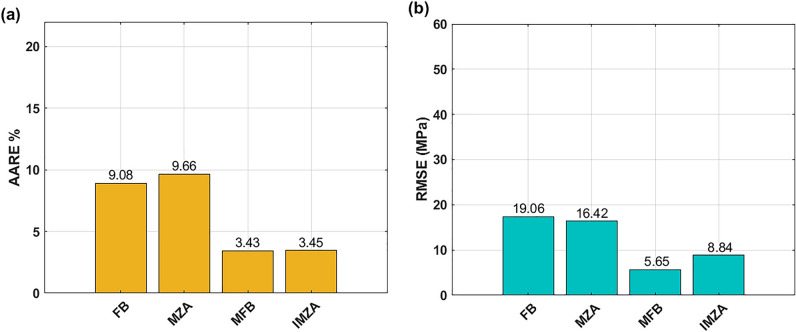
(**a**) AARE for FB, MZA, MFB, and IMZA models, (**b**) RMSE for FB, MZA, MFB, and IMZA models.

Calculated values of AARE (see Eq. (35)) and RMSE (see Eq. (36)) are plotted using histograms in Fig. 16. As it can be seen, the MFB model gives the best AARE value of 3.34%, and the IMZA model comes next with AARE value of 3.54%, while the FB and MZA models give higher AARE values of 9.08 and 9.66% respectively (cf. Fig. 16a). Concerning RMSE, the MFB model gives the best RMSE with a value of 5.64 MPa, and the IMZA comes next with a value of 8.84 MPa, while the MZA and FB models give 16.42 and 19.06 MPa respectively (cf. Fig. 16b).

### Validation of the models

In this subsection, the applicability of the FB, MZA, MFB, and IMZA models are investigated by studying R, AARE, and RMSE of two other titanium-based alloys SP700 and TC4. The hot deformation behavior of the two alloys is considered in^59^ and^60^ respectively.

**Figure 17:**
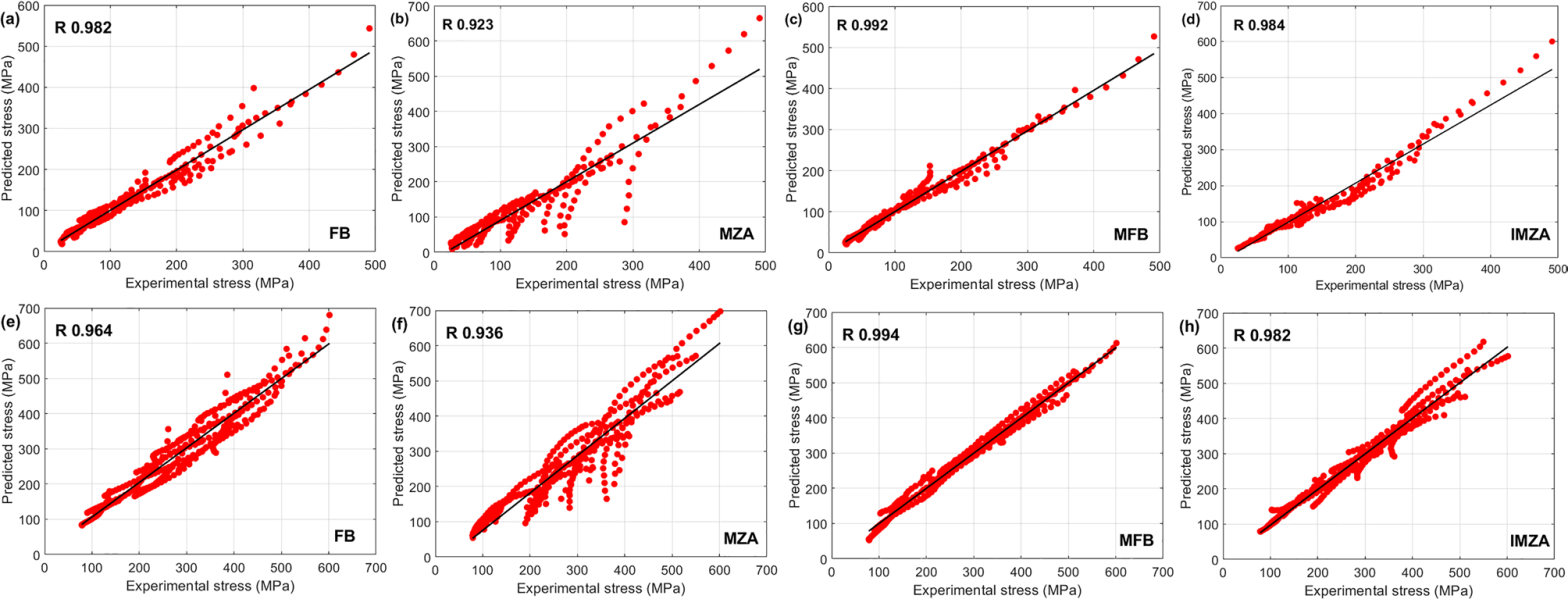
Correlation of experimental stresses and predicted stresses by (**a**) FB, (**b**) MZA, (**c**) MFB, and (**d**) IMZA models for SP700 and (**e**) FB, (**f**) MZA, (**g**) MFB, and (**h**) IMZA models for TC4.

Figure 17 shows the correlation between experimental stresses and predicted stresses by FB, MZA, MFB, and IMZA models for the SP700 alloy (Fig. 17a, b, c, d) and TC4 alloy (Fig. 17e, f, g, h). It is shown that the MFB model gives the best R values for both alloys with values of 0.994 and 0.992 for TC4 and SP700 respectively. The IMZA model gives the second good values with an R of 0.984 and 0.982 for SP700 and TC4 respectively. On the other hand, the lowest R values are obtained by the MZA model with values of 0.936 and 0.923 for TC4 and SP700 respectively.

**Figure 18:**
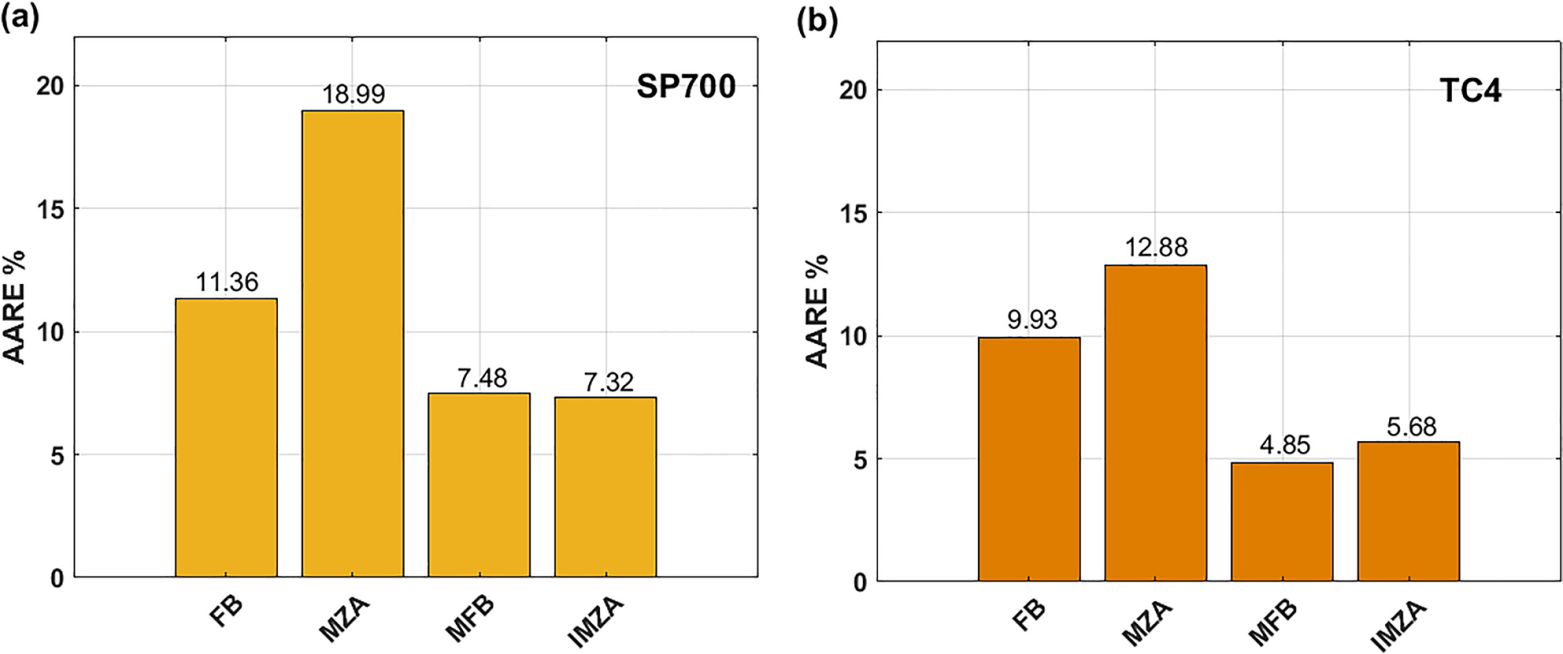
(**a**) AARE of FB, MZA, MFB, and IMZA models for SP700 alloy, (**b**) AARE of FB, MZA, MFB, and IMZA models for TC4 alloy.

Figure 18 shows a histogram for the AARE values of the FB, MZA, MFB, and IMZA models for the SP700 alloy (see Fig. 18a) and for the TC4 alloy (see Fig. 18b). The lowest AARE value is 7.32%, which is obtained by the IMZA model for SP700 alloy, and the second value is related to the MFB model with a value of 7.48%, while the high values 11.36% and 18.99% are obtained with FB and MZA models respectively. With respect to TC4 alloy, the lowest AARE value 4.85% is associated with the MFB model, and the second lowest AARE value 5.68% is related to the IMZA model, while the high values 9.93% and 12.88% are associated to the FB and MZA models respectively.

**Figure 19:**
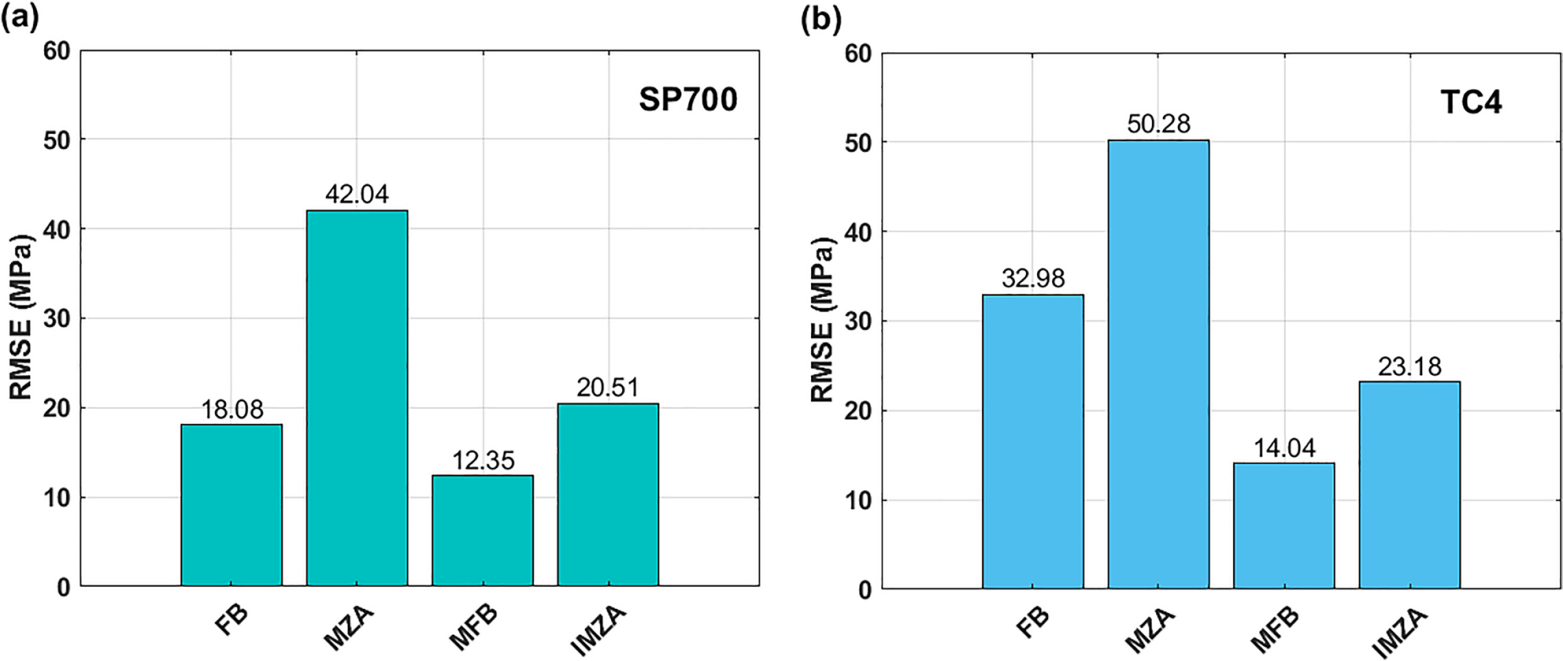
(**a**) RMSE of FB, MZA, MFB, and IMZA models for SP700 alloy, (**b**) RMSE of FB, MZA, MFB, and IMZA models for TC4 alloy.

A histogram for the RMSE of the FB, MZA, MFB, and IMZA models for the SP700 alloy and TC4 alloy is shown in Fig. 19. Considering SP700 alloy, the lowest RMSE value 12.35 MPa is associated to the MFB model, and the second lowest value 18.08 MPa is related to the FB model, while the high values 20.51 MPa and 42.04 MPa are associated to the IMZA and MZA models respectively (cf. Fig. 19a). Considering TC4 alloy, the lowest RMSE value 14.04 MPa is associated to the MFB model, and the second lowest value 23.18 MPa is related to the IMZA model, while the high values 32.98 MPa and 50.28 MPa are associated to the FB and MZA models respectively (cf. Fig. 19b).

## Conclusion

In this study, modified Fields-Backofen and Zerilli-Armstrong constitutive models are presented to predict the hot deformation behavior of titanium-based alloys. To find the best performance, the modified models along with the original Fields-Backofen model and another modification of the Zerilli-Armstrong model are investigated by studying the flow behavior of Ti55531 alloy during hot deformation. In addition, the models are validated by studying its predictability on other two titanium-based alloys namely SP700 and TC4. Conclusions can be summarized as:

- The MFB model gives the best R value among the four models for the Ti53331, TC4, and SP700 alloys with values of 0.996, 0.994, and 0.992 respectively. The IMZA model gives the second-best values of 0.992, 0.982, and 0.984 respectively. On the contrary, the original FB and the MZA give the lowest values of R that ranges from 0.923 to 0.982 for the three alloys.
- Considering the AARE, the MFB model along with the IMZA model give the lowest AARE values of 3.34% and 3.52% for the Ti55531 alloy and 7.48% and 7.32% for the SP700 alloy respectively. Regarding the TC4 alloy, the best AARE value of 4.85% is associated to the MFB model, while the second-best AARE value of 5.68% is related to the IMZA. On the other hand, the original FB and the MZA give the high AARE values that range from 9.93% to 18.99% for the three alloys.
- The best RMSE values of 5.65 MPa, 12.35 MPa, and 14.04 MPa are associated to the MFB model for the Ti55531, SP700, and TC4 respectively. The IMZA gives the second-best RMSE values of 8.84 MPa and 23.18 MPa for the Ti55531 and TC4 respectively, while the FB gives the second-best value of RMSE of 18.08 MPa for the SP700 alloy. Conversely, the original FB and the MZA models give the high values that ranges from 16.42 MPa to 50.28 MPa for the three alloys.

In sum, among the four models, the MFB model gives the best performance and the IMZA comes in the second-best place with a very good accuracy, while the original FB model and the MZA model come in the third and fourth best place with not accurate predictions.

## Data Availability

The dataset used and analysed during the current study is available from the corresponding author on reasonable request.

## References

[1] Wang M, Zhou J, Yin Y, Nan H, Xue P, Tu Z (2017). Hot deformation behavior of the Ti6Al4V alloy prepared by powder hot isostatic pressing. J. Alloy. Compd..

[2] Wang Z, Tan Y, Li N (2023). Powder metallurgy of titanium alloys: A brief review. J. Alloy. Compd..

[3] Cam G, Flower HM, West DRF (1991). Constitution of Ti–Al–C alloys in temperature range 1250–750°C. Mater. Sci. Technol..

[4] Gürel C, Helmut C, Gerling R, Koçak M (1999). Diffusion bonding of fine grained γ-TiAl sheet. Int. J. Mater. Res..

[5] Çam G, İpekoğlu G, Bohm KH, Koçak M (2006). Investigation into the microstructure and mechanical properties of diffusion bonded TiAl alloys. J. Mater. Sci..

[6] Banerjee D, Williams JC (2013). Perspectives on titanium science and technology. Acta Mater..

[7] Boyer RR, Briggs RD (2013). The use of β titanium alloys in the aerospace industry. J. Mater. Eng. Perform..

[8] Jones NG, Jackson M (2011). On mechanism of flow softening in Ti–5Al–5Mo–5V–3Cr. Mater. Sci. Technol..

[9] Li C, Huang L, Zhao M, Guo S, Su Y, Li J (2022). Systematic analysis of the softening mechanism and texture evolution of Ti–6Cr–5Mo–5V–4Al alloy during hot compression in α+ β phase region. Mater. Sci. Eng. A.

[10] Shi S, Ge J, Lin YC, Zhang X, Zhou K (2022). High-temperature deformation behavior and recrystallization mechanism of a near beta titanium alloy Ti-55511 in β phase region. Mater. Sci. Eng. A.

[11] Shokry A (2017). A modified Johnson-Cook model for flow behavior of alloy 800H at intermediate strain rates and high temperatures. J. Mater. Eng. Perform..

[12] Shokry A, Gowid S, Kharmanda G (2021). An improved generic Johnson-Cook model for the flow prediction of different categories of alloys at elevated temperatures and dynamic loading conditions. Mater. Today Commun..

[13] Luo Y, Shi C, Xu H (2023). Microstructure evolution and hot deformation characteristics of 15Cr-22Ni iron-base superalloy. J. Alloy. Compd..

[14] Sim KH, Ri YC, Jo CH, Kim OJ, Kim RS, Pak H (2023). Modified Zerilli-Armstrong and Khan-Huang-Liang constitutive models to predict hot deformation behavior in a powder metallurgy Ti-22Al-25Nb alloy. Vacuum.

[15] Chen XM, Ning MT, Hu HW, Lin YC, Zhou XJ, Zhang J, Lu XZ, Chen JM, Liu YX (2023). Characterization of hot deformation behavior and optimization of hot workability for GH4698 superalloy. Mater. Charact..

[16] Lin YC, Chen XM (2011). A critical review of experimental results and constitutive descriptions for metals and alloys in hot working. Mater. Des..

[17] Shokry A, Gowid S, Mulki H, Kharmanda G (2023). On the prediction of the flow behavior of metals and alloys at a wide range of temperatures and strain rates using Johnson-Cook and modified Johnson–Cook-based models: A review. Materials.

[18] Khan AS, Huang S (1992). Experimental and theoretical study of mechanical behavior of 1100 aluminum in the strain rate range 10^–5^−10^4^s^−1^. Int. J. Plast..

[19] Johnson, G. R. & Cook, W. H. A constitutive model and data for metals subjected to large strains, high strain rates and high temperatures. In *Proceedings of the 7th International Symposium on Ballistics*, (1983).

[20] Fields, D. S., & Backofen, W. A. Determination of strain hardening characteristics by torsion testing. In *Proc. ASTM *(Vol. 57, No. 0, pp. 1259–1272), (1957).

[21] Zerilli FJ, Armstrong RW (1987). Dislocation-mechanics-based constitutive relations for material dynamics calculations. J. Appl. Phys..

[22] Goetz RL, Seetharaman V (1998). Modeling dynamic recrystallization using cellular automata. Scr. Mater..

[23] Preston DL, Tonks DL, Wallace DC (2003). Model of plastic deformation for extreme loading conditions. J. Appl. Phys..

[24] Shokry A, Gowid S, Kharmanda G, Mahdi E (2019). Constitutive models for the prediction of the hot deformation behavior of the 10% Cr steel alloy. Materials.

[25] Shokry A, Gowid S, Youssef SS (2022). Modeling the flow behavior of Haynes 214 superalloy during hot deformation using mathematical and artificial intelligence-based models. Mater. Today Commun..

[26] Hu DC, Wang L, Wang N, Chen MH, Wang HJ (2022). Hot tensile deformation behaviors of TA32 titanium alloy based on back-propagation neural networks and three-dimensional thermal processing maps. J. Mater. Res. Technol..

[27] Moghadam NN, Serajzadeh S (2023). Warm and hot deformation behaviors and hot workability of an aluminum-magnesium alloy using artificial neural network. Mater. Today Commun..

[28] Liu J, Zeng W, Lai Y, Jia Z (2014). Constitutive model of Ti17 titanium alloy with lamellar-type initial microstructure during hot deformation based on orthogonal analysis. Mater. Sci. Eng. A.

[29] Yu W, Li Y, Cao J, Yang Z, Zhang J, Lang S (2021). The dynamic compressive behavior and constitutive models of a near α TA23 titanium alloy. Mater. Today Commun..

[30] Gao S, Sang Y, Li Q, Sun Y, Wu Y, Wang H (2022). Constitutive modeling and microstructure research on the deformation mechanism of Ti-6Al-4V alloy under hot forming condition. J. Alloy. Compd..

[31] Jiang F, Fei L, Jiang H, Zhang Y, Feng Z, Zhao S (2023). Constitutive model research on the hot deformation behavior of Ti6Al4V alloy under wide temperatures. J. Mater. Res. Technol..

[32] Tsao LC, Wu HY, Leong JC, Fang CJ (2012). Flow stress behavior of commercial pure titanium sheet during warm tensile deformation. Mater. Des..

[33] Li X, Guo G, Xiao J, Song N, Li D (2014). Constitutive modeling and the effects of strain-rate and temperature on the formability of Ti–6Al–4V alloy sheet. Mater. Des..

[34] Wen Y, Zhang T, Yan W, Chen Y, Wang G (2021). Mechanical response of porcine hind leg muscles under dynamic tensile loading. J. Mech. Behav. Biomed. Mater..

[35] Huang X, Wang B, Zang Y, Ji H, Guan B, Li Y, Tang X (2020). Constitutive relationships of 21–4 N heat-resistant steel for the hot forging process. J. Mater. Res. Technol..

[36] Cai Z, Ji H, Pei W, Wang B, Huang X, Li Y (2019). Constitutive equation and model validation for 33Cr23Ni8Mn3N heat-resistant steel during hot compression. Res. Phys..

[37] Ahmedabadi PM, Kain V (2022). Constitutive models for flow stress based on composite variables analogous to Zener-Holloman parameter. Mater. Today Commun..

[38] Ji G, Li L, Qin F, Zhu L, Li Q (2017). Comparative study of phenomenological constitutive equations for an as-rolled M50NiL steel during hot deformation. J. Alloy. Compd..

[39] Li F, Zhu C, Li S, Jiang H, Zhang P, Yang R, Zhao S (2022). A comparative study on modified and optimized Zerilli-Armstrong and arrhenius-type constitutive models to predict the hot deformation behavior in 30Si2MnCrMoVE steel. J. Mater. Res. Technol..

[40] Zhang H, Wen W, Cui H, Xu Y (2009). A modified Zerilli-Armstrong model for alloy IC10 over a wide range of temperatures and strain rates. Mater. Sci. Eng. A.

[41] Mirzaie T, Mirzadeh H, Cabrera JM (2016). A simple Zerilli-Armstrong constitutive equation for modeling and prediction of hot deformation flow stress of steels. Mech. Mater..

[42] Gurusamy MM, Rao BC (2017). On the performance of modified Zerilli-Armstrong constitutive model in simulating the metal-cutting process. J. Manuf. Process..

[43] Samantaray D, Mandal S, Borah U, Bhaduri AK, Sivaprasad PV (2009). A thermo-viscoplastic constitutive model to predict elevated-temperature flow behaviour in a titanium-modified austenitic stainless steel. Mater. Sci. Eng. A.

[44] Murugesan M, Jung DW (2019). Two flow stress models for describing hot deformation behavior of AISI-1045 medium carbon steel at elevated temperatures. Heliyon.

[45] Trimble D, Agarwal A, McDonnell D, Barron S, Ahearne E, O’Donnell GE (2020). Finite element simulation of orthogonal machining of biomedical grade co–cr–mo alloy. CIRP J. Manuf. Sci. Technol..

[46] Lewis J, Pasco J, McCarthy T, Chadha K, Harding M, Aranas C (2022). High strain rate and high temperature mechanical response of additively manufactured alloy 625. J. Manuf. Process..

[47] Shokry A (2019). On the constitutive modeling of a powder metallurgy nanoquasicrystalline Al93Fe3Cr2Ti2 alloy at elevated temperatures. J. Braz. Soc. Mech. Sci. Eng..

[48] Yu LIU, Ming LI, Ren XW, Xiao ZB, Zhang XY, Huang YC (2020). Flow stress prediction of Hastelloy C-276 alloy using modified Zerilli− Armstrong, Johnson− Cook and Arrhenius-type constitutive models. Trans. Nonferrous Metals Soc. China.

[49] Yu R, Li X, Li W, Chen J, Guo X, Li J (2021). Application of four different models for predicting the high-temperature flow behavior of TG6 titanium alloy. Mater. Today Commun..

[50] Singh G, Chakraborty P, Tiwari V (2023). A comparative study of different constitutive models to predict the dynamic flow behaviour of a homogenised AT61 magnesium alloy. Structures.

[51] El Kadiri H, Wang L, Ozkan GH, Suri P, Park SJ, Hammi Y, German RM (2009). Development of a Ti-based alloy: Design and experiment. JOM.

[52] Liang HQ, Nan Y, Ning YQ, Li H, Zhang JL, Shi ZF, Guo HZ (2015). Correlation between strain-rate sensitivity and dynamic softening behavior during hot processing. J. Alloy. Compd..

[53] Xiang Y, Xiang W, Yuan W (2023). Flow softening and microstructural evolution of near β titanium alloy Ti-55531 during hot compression deformation in the α+ β region. J. Alloy. Compd..

[54] Li T, Lu Y, Li Z, Wang T, Li T (2022). Hot deformation behavior and microstructure evolution of non-equimolar Ti2ZrHfV0. 5Ta0. 2 refractory high-entropy alloy. Intermetallics.

[55] Hu X, Wang Z, Wang L, Chen C, Zhang F, Zhang W (2022). Effect of pre-deformation on hot workability of super austenitic stainless steel. J. Mater. Res. Technol..

[56] Li X, Le Q, Li D, Wang P, Jin P, Cheng C, Cheng X, Ren L (2021). Hot tensile deformation behavior of extruded LAZ532 alloy with heterostructure. Mater. Sci. Eng. A.

[57] Che B, Lu L, Kang W, Luo J, Ma M, Liu L (2021). Hot deformation behavior and processing map of a new type Mg-6Zn-1Gd-1Er alloy. J. Alloy. Compd..

[58] Kotkunde N, Deole AD, Gupta AK, Singh SK (2014). Comparative study of constitutive modeling for Ti–6Al–4V alloy at low strain rates and elevated temperatures. Mater. Des..

[59] Qiu Q, Wang K, Li X, Wang J, Gao X, Zhang K (2021). Hot deformation behavior and processing parameters optimization of SP700 titanium alloy. J. Mater. Res. Technol..

[60] Xiawei YANG, Yanying WANG, Xiurong DONG, Chong PENG, Baijin JI, Yaxin XU, Wenya LI (2021). Hot deformation behavior and microstructure evolution of the laser solid formed TC4 titanium alloy. Chin. J. Aeronaut..

